# Multimodal Image-Based Indoor Localization with Machine Learning—A Systematic Review

**DOI:** 10.3390/s24186051

**Published:** 2024-09-19

**Authors:** Szymon Łukasik, Szymon Szott, Mikołaj Leszczuk

**Affiliations:** 1 Systems Research Institute Polish Academy of Sciences, 01-447 Warszawa, Poland; 2 AGH University of Krakow, 30-059 Krakow, Poland; szymon.szott@agh.edu.pl (S.S.); mikolaj.leszczuk@agh.edu.pl (M.L.)

**Keywords:** computer vision, data fusion, indoor localization, machine learning, positioning

## Abstract

Outdoor positioning has become a ubiquitous technology, leading to the proliferation of many location-based services such as automotive navigation and asset tracking. Meanwhile, indoor positioning is an emerging technology with many potential applications. Researchers are continuously working towards improving its accuracy, and one general approach to achieve this goal includes using machine learning to combine input data from multiple available sources, such as camera imagery. For this active research area, we conduct a systematic literature review and identify around 40 relevant research papers. We analyze contributions describing indoor positioning methods based on multimodal data, which involves combinations of images with motion sensors, radio interfaces, and LiDARs. The conducted survey allows us to draw conclusions regarding the open research areas and outline the potential future evolution of multimodal indoor positioning.

## 1. Introduction

Positioning (or localisation) is the determination of the absolute or relative placement of objects in a three-dimensional space. In outdoor environments, this goal is usually achieved through the analysis of radio signals from satellites forming a global navigation satellite system (GNSS). Such systems achieve an accuracy of around 10 m [1]. This ubiquitous technology has led to the widespread adoption of multiple location-based services (LBSs) such as turn-by-turn navigation or asset tracking [2]. So far, similar success has not been achieved in indoor environments, where signal propagation prevents the use of a GNSS and a higher degree of location accuracy is required [3]. Thus, despite efforts from researchers and industry, a single solution for indoor positioning has not yet emerged.

One of the major obstacles to achieving precise indoor localisation is achieving the high positioning accuracy necessary to provide services, such as navigating to a product on a shelf or providing secure office room access. Meanwhile, deployment costs need to remain low since separate instalments are required in each building. An interesting approach to meeting these criteria is the use of multiple sources of information already available. In much the same way that outdoor smartphone positioning has better accuracy when multiple data sources are combined (e.g., GNSS information with cell tower triangulation), indoor positioning can similarly benefit from such data fusion.

**Figure 1:**
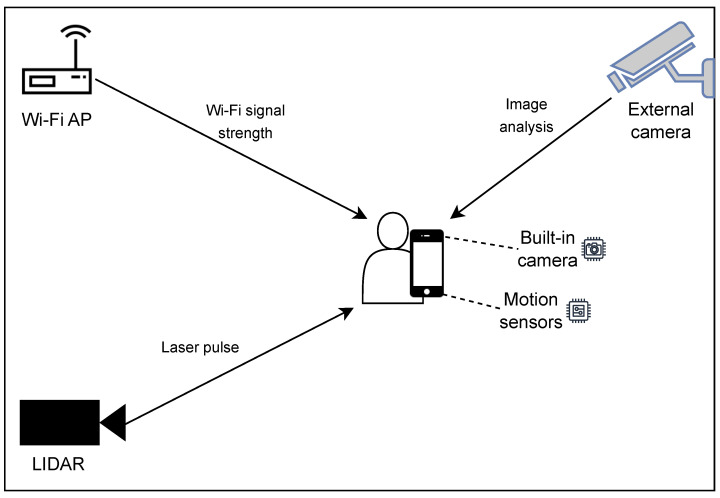
Exemplary input data for multimodal indoor positioning: image analysis from internal and external cameras, motion sensor data, signal strength of beacons transmitted by Wi-Fi APs, and LiDAR measurements.

Figure 1 presents examples of typical signals that are available for indoor positioning and that can be combined in a multimodal localisation solution. First, we have visual signals that can originate from external (surveillance-type) cameras or internal (built-in) cameras. Second, we have motion sensors, which are readily available in all smartphones. Third, we have radio signals of various technologies, such as from Wi-Fi access points (APs) [4,5], and Bluetooth or ultra-wideband (UWB) beacons [6,7]. Finally, we may have dedicated light detection and ranging (LiDAR) measurement units. Thus, the next challenge that arises is how to analyse the input data and successfully combine multiple sources.

Machine learning (ML) methods are currently being successfully applied in a variety of fields, such as computer vision [8,9] and improving network performance [10]. For multimodal indoor positioning, they have two important areas of application. First, they can extract features from signals to provide useful information, for example, scene recognition from camera imagery. In this case, typically neural networks (NNs) are used. Second, they can combine location information provided by several sources to reduce the uncertainty of positioning. Here, methods such as Kalman filter (KF) or particle filter (PF) are typically used. Thus, ML arises as an important element of an indoor positioning system.

Based on the above reasoning, we conduct a survey of research on indoor localisation that uses machine learning to analyse and combine data from multiple sources, one of which is image-based. We focus on this particular aspect because camera-based imagery is readily available (both in-room and built-in cameras) and can provide additional information to other indoor positioning systems. Formally, our survey is a ‘systematic review’ [11] as explained in Section 2. Although there have been multiple indoor positioning surveys, including a meta-survey [12], we justify our contribution by noting that (a) there has not been a survey of a scope similar to ours and (b) most of the recent articles have not yet been cited by any survey. Furthermore, our unique perspective allows us to draw relevant conclusions in this vibrant field of research.

The remainder of this paper is organised as follows; After describing our methodology (Section 2), we review the papers, which we have classified as follows: First, we describe papers relying solely on camera-based information (although still adhering to the multimodal principle) in Section 3. Then, in Section 4, we focus on papers that add information from motion sensors. Next, we analyse methods that rely on a radio interface (Section 5), LiDAR (Section 6), or other sensors (Section 7) for multimodal indoor localisation. Finally, we summarise our findings and outline future research directions in Section 8.

## 2. Methodology

Our paper’s methodological layer follows the good practices of systematic reviews. This type of review aims to bring evidence together to answer a predefined research question. This involves identifying all primary research relevant to the defined review question, critically evaluating this research, and synthesising the findings [11]. To achieve that goal, in the framework of our research, we have collected the scientific material using the Scopus (https://www.scopus.com/, accessed on 1 July 2024) search engine. We have used the following search query to identify relevant studies:

(multimodal OR fusion) AND indoor AND (localization OR location OR positioning) AND (vision OR camera OR image) AND learning

The construction of the query allowed us to capture the alternative use of similar terms (e.g., vision/camera/image). The search was performed within article title, abstract, and keywords, using a filter on the content type, allowing to include articles, conference papers, book chapters, and data papers. The search was conducted in April 2024. At this stage of data acquisition, 115 documents were collected. Subsequent screening of manuscripts for relevance to the scope of our research resulted in the removal of 73 documents from the collected set. Of the remaining 42 documents, 22 were journal articles and 20 conference papers. We have divided the documents according to the signals used for data fusion into the following categories:

- fusion of signals collected from camera sensors;
- fusion of image data with motion signals;
- fusion of image data with radio signals;
- fusion of image data with LiDAR signals;
- fusion of image data with signals from other sensors, not considered elsewhere.

**Figure 2:**
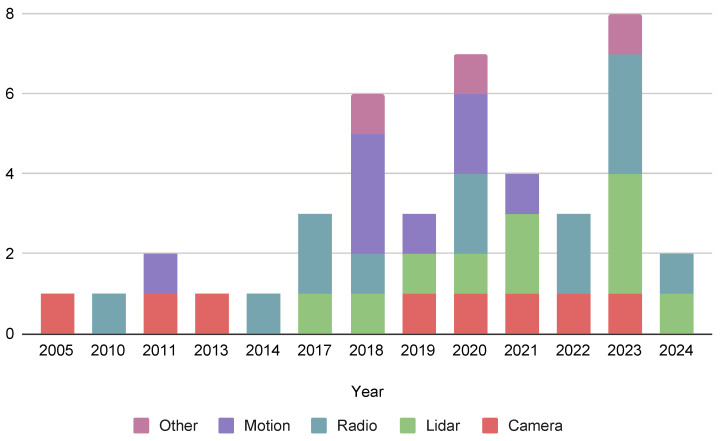
Timeline and distribution of collected publications across different data fusion schemes.

Figure 2 shows the distribution of the materials among the distinguished fusion schemes. The following chapters will cover the analysed materials in more detail.

## 3. Camera-Based Positioning

The evolution of camera-based sensors has greatly improved various fields, from indoor surveillance and behaviour analysis to advanced navigation systems. This section explores advances in camera-based technologies through a chronological review of key studies, starting from a comprehensive survey conducted in 2019 and covering subsequent innovations.

Bai et al. [13] in the aforementioned study provided a comprehensive survey on camera-based localisation techniques that use deep learning. Their work emphasised the effectiveness of convolutional neural networks (CNNs) in processing visual data for accurate indoor positioning and navigation, setting a solid foundation for future research in this domain. This study underscored the potential of deep learning to significantly improve the capabilities of camera-based sensor applications in complex indoor and outdoor environments. By highlighting the various deep learning models used for camera pose estimation, this survey paved the way for further research into advanced ML techniques to improve localisation accuracy. In addition, the survey discussed the integration of multiple sensor modalities, such as combining visual information from red-green-blue (RGB) and red-green-blue-depth (RGB-D) cameras with depth data. This multimodal approach, called sensor fusion, enhances localisation performance by leveraging the strengths of different types of data to provide more robust and accurate positioning solutions. The use of sensor fusion techniques, including the integration of visual and depth information, exemplifies the growing trend towards using multimodal data to overcome the limitations of single-sensor systems and improve the overall reliability and accuracy of localisation systems.

Based on these findings, Narayanan et al. [14] introduced ProxEmo, an innovative algorithm designed for emotion prediction using camera footage. This system combines visual data with gait analysis using a multiview skeleton graph convolution-based model to classify emotions. By integrating proxemic fusion with LiDAR data, ProxEmo informs robot navigation decisions, allowing robots to adjust their paths based on the perceived emotions of nearby pedestrians. This approach enhances human-robot interaction by enabling socially aware robotic behaviours, demonstrating the potential of multimodal systems that fuse visual and spatial data for dynamic emotional recognition and response.

The progress in camera-based sensor technology continued with Zhang and Leonard [15], who proposed a novel approach to dense mapping using camera sensors, focussing on improving the accuracy of simultaneous localization and mapping (SLAM) for robotic navigation and environmental mapping. Their method fused depth information from a single RGB image with learnt outlier masks to improve robustness in dynamic environments. By utilising CNNs for pose estimation and generating an outlier mask to handle unreliable regions caused by occlusions or dynamic objects, this approach improved SLAM algorithms. The integration of semantic segmentation images with depth data enriched the environmental model, making the system more reliable for complex indoor and outdoor operations.

In the same vein, Yang et al. [16] described the signed distance function SLAM (SDF-SLAM) technique, which leverages camera data to enhance real-time SLAM performance. By integrating depth estimation and semantic segmentation, this method significantly improves navigation and mapping capabilities, showcasing advancements in camera sensor technology for autonomous systems. The deep-learning-based model processes monocular camera images to generate three-dimensional semantic maps, providing a more detailed and informative understanding of the environment. This multimodal fusion of depth and semantic data leads to more precise point cloud generation and camera pose estimation, especially in complex indoor settings where traditional SLAM techniques may falter.

In 2011, Md Mahfujur Rahman and El Saddik [17] developed a pioneering end-to-end emotion recognition system that uses camera sensors to capture physical interactions and facial expressions, providing real-time feedback crucial for human-computer interaction and behavioural studies. The interactive engagement of this system with users represented a significant advancement in camera-based emotion recognition, with usability studies highlighting high user engagement and practical applications across various domains. The integration of visual data with infrared (IR) sensors and accelerometer readings improved the accuracy and reliability of emotion detection, allowing for a more precise interpretation of emotional states through multimodal data fusion. This comprehensive approach is essential for real-time emotion recognition, ensuring robust and contextually aware user experiences.

Narayanan et al. [18] explored the application of omnidirectional cameras in mobile robots for learning behavioral dynamics. These 360-degree cameras capture comprehensive data in dynamic environments, improving the robot’s ability to understand and interact with its surroundings. Their use enables more flexible and robust robotic systems that can adapt to changing environments and learn from demonstrations. The experiments demonstrated significant improvements in navigation accuracy and obstacle avoidance, underscoring the practical benefits of omnidirectional cameras. By integrating visual data with other sensory inputs, such as sonar or LiDAR, using image segmentation and Gaussian mixture models (GMMs), the system created a comprehensive map of the environment, improving the robot’s ability to navigate and avoid obstacles in complex, dynamic settings.

Earlier contributions to the field include the work of Petrushin et al. [19], who in 2005 proposed the multiple sensor indoor surveillance (MSIS) system, which integrates web cameras, infrared badge identifier (ID) systems, and pan-tilt-zoom (PTZ) cameras to enhance indoor surveillance. The combination of these sensor inputs improved the accuracy and reliability of the monitoring activities, laying the foundation for future multisensor surveillance systems. Using Bayesian inference and self-organising maps (SOMs) for event clustering and classification, the system demonstrated early innovation in multisensor integration. The fusion of visual data with positional tracking from infrared badges allowed precise individual tracking, while PTZ cameras provided dynamic coverage. This multimodal integration improved person location and event detection, creating a robust system capable of maintaining accurate surveillance even with incomplete or noisy data.

More recently, in 2023, Wen et al. [20] presented an innovative indoor navigation method using camera sensors integrated with deep learning techniques for precise positioning and navigation. This approach, combining advantage actor-critic (A2C) and cross-modal attention techniques, significantly improved navigation success rates and reduced trajectory lengths, showcasing the effectiveness of advanced ML algorithms in autonomous navigation systems. By merging visual data from cameras with language instructions in a vision and language navigation (VLN) task, the system uses cross-modal feature fusion and attention mechanisms to align visual and language features. This multimodal integration allows the agent to better interpret and execute navigation tasks, while reinforcement learning optimises action selection, making the system highly effective in complex indoor environments.

Table 1 underscores the progressive advancements in camera-based sensor technologies and their transformative impact in various domains.

## 4. Camera-Based Positioning with Motion Sensors

In recent years, there has been significant progress in the development of indoor positioning systems that use camera-based technology in combination with motion sensors. These systems aim to provide precise localisation in environments where traditional positioning technologies, such as global positioning system (GPS), are ineffective. By integrating visual data with inertial measurements, researchers have achieved notable improvements in accuracy and robustness, addressing challenges such as signal degradation, occlusions, and dynamic changes in the environment.

The integration of these technologies has the potential to transform various applications, ranging from navigation in complex indoor environments to emergency response and augmented reality experiences. This section discusses the methodologies and results of several studies that have contributed to this rapidly evolving field (Table 2).

A key advantage of combining these two types of sensors is their complementary nature. Cameras provide rich contextual information and high spatial resolution, while inertial sensors offer high temporal resolution and robustness to environmental changes. This combination allows for more accurate and reliable positioning, even in challenging indoor environments. The studies covered in this section span a range of methodologies, applications, and innovations, showcasing the versatility and potential of camera-based positioning systems.

Furthermore, the use of advanced ML techniques has further enhanced the capabilities of these systems. Deep learning models, in particular, have been instrumental in processing complex data generated by cameras and motion sensors, enabling more sophisticated and accurate positioning solutions. As technology continues to evolve, the integration of camera and motion sensor data is expected to play an increasingly critical role in various fields.

Yan et al. [21] propose a system for indoor pedestrian dead reckoning that integrates visual tracking and map information. Using inertial sensors and surveillance cameras, their approach addresses the problem of drift accumulation in position estimation by supplementing inertial navigation for a hand-held smartphone with vision data. Inertial sensors, readily available in smartphones, can be used for pedestrian dead reckoning (PDR) to estimate the current location by tracking user movements from a previously known position. However, this approach suffers from drift in position estimation over time. To mitigate this, Yan et al. [21] integrate vision data from surveillance cameras with inertial data. A CNN is employed to detect pedestrians in the recorded images, significantly enhancing the localization accuracy. The system, tested in a real-world office setting using smartphones, demonstrated enhanced localization accuracy through the integration of visual and inertial data. The deep learning model used in this study is able to learn complex patterns from the sensor data, providing a robust solution to the problem of drift accumulation in inertial systems.

In another study, Turaga and Ivanov [22] describes the “Diamond Sentry” system, which combines cameras and motion sensors for real-time monitoring of indoor spaces. This system effectively detects and responds to events using sensor data and camera feeds, providing accurate activity recognition and event analysis. The minimal commitment strategy maximises the utility of video data, improving the effectiveness of the system in event detection. This approach reduces the computational burden by processing only data when necessary, making it suitable for real-time applications in environments such as offices and public spaces.

Yan et al. [23] further explore vision-aided PDR, utilizing micro-electro-mechanical systems (MEMS) and charge-coupled device (CCD) cameras to achieve a mean accuracy improvement of 11% over uncalibrated inertial systems. Their approach incorporates deep learning and geographic information systems (GISs), providing a more accurate and continuous positioning service. The integration of GIS allows the system to correlate sensor data with spatial information, enhancing the overall accuracy and reliability of the positioning system. This study also highlights the potential of using publicly available GIS data to improve indoor navigation solutions.

The fusion of inertial and visual information is also addressed by Xu et al. [24], who introduce a method that combines extreme learning machines and Dempster-Shafer evidence theory, significantly enhancing localisation accuracy in challenging scenarios, including occlusions and light changes. The system is tested in a college building, demonstrating real-time processing capabilities and robustness. The proposed method is particularly effective in environments with dynamic changes, as it can adapt to new data and update its predictions accordingly.

Li and Sridharan [25] propose a method that enhances the localization and navigation of mobile robots by integrating local image gradient cues with temporal visual cues and range sensor data. The approach employs a combination of maximally stable extremal regions (MSER) and scale-invariant feature transform (SIFT) for object detection and tracking. This visual information is fused with range data using a Kalman filter, which allows the robot to navigate safely in dynamic environments. The system was tested on a humanoid robot in various indoor settings, demonstrating robust obstacle avoidance and improved localization accuracy. This methodology highlights the effectiveness of combining different types of sensory data to improve the overall performance of mobile robots in complex environments.

A calibration-free visual-inertial fusion technique using deep convolutional recurrent neural networks (DCRNNs) is proposed by Soroush Sheikhpour and Atia [26] (2019). This method learns the navigation parameters and dynamics of the system directly from the data, eliminating the need for manual calibration. The system shows robust pose estimation in both indoor and outdoor environments. The DCRNN model is able to capture temporal dependencies in sensor data, providing a more accurate and reliable positioning solution. This study demonstrates the potential of using deep learning to simplify the deployment of indoor navigation systems by reducing the need for extensive calibration.

Dai et al. [27] present an indoor positioning system using millimeter-wave radar and inertial sensors, designed for visually-degraded environments. Their system successfully tracks users in real-time, even in poor lighting conditions, by fusing radar and inertial data. The method employs convolutional and recurrent neural networks, demonstrating good accuracy and resilience in a dark apartment setting. This approach is particularly useful in environments where visual data may be unreliable or unavailable, such as during power outages or in smoke-filled areas.

Finally, Hu and Liao [28] propose a deep learning and sensor fusion approach for the location of cameras in real time. Their system achieves high accuracy and efficiency with an orientation error of 2.5179 degrees and a position error of 0.0222 metres. The method is tested in an indoor environment, demonstrating its potential for augmented reality (AR) applications. The use of deep learning models allows the system to process large amounts of data quickly, making it suitable for real-time applications such as AR navigation and interactive gaming.

**Table 2:** Summary of camera-based positioning with motion sensors studies.

Reference	Year	Input Data	Localized Object	Metrics	ML Method	Outcome
Li and Sridharan [25]	2010	Surveillance cameras, range sensors	Robot	Obstacle avoidance accuracy, localization error	MSER, SIFT, Kalman Filter	Robust localization and tracking of obstacles in dynamic environments, achieving obstacle avoidance with an average distance error of 21.7 cm and bearing error of 1.8° using fused local and temporal visual cues with range sensors
Turaga and Ivanov [22]	2011	Surveillance cameras, motion sensors	People, event	Event detection	General algorithms	Efficient monitoring, maximizing video utility by reducing data overheads by 60% through sensor-guided camera control, leading to real-time, scalable surveillance in indoor environments
Yan et al. [21]	2018	Surveillance cameras, motion sensors	Smartphone	Localization error (RMSE)	CNN	IImproved localization with visual tracking, achieving an RMSE of 0.73 m after calibration, with a 10% reduction in drift over long corridors by combining pedestrian dead reckoning and visual tracking
Yan et al. [23]	2018	Surveillance cameras, GIS, motion sensors	People	Accuracy improvement over uncalibrated inertial systems	Faster R-CNN	More accurate, continuous positioning, with localization errors reduced by up to 69.7% through visual tracking calibration and map information integration, achieving RMSEs as low as 0.51 m for Android and 0.56 m for iOS
Xu et al. [24]	2018	Surveillance cameras, motion sensors	Env.	Localization accuracy (Euclidean distance, RMSE)	ELM, D-S evidence theory	Enhanced localization in challenging scenarios, with a mean error as low as 0.07 m and RMSE reduced to 0.10 m under interference conditions, using ELM and Dempster-Shafer fusion of visual and inertial data
Soroush Sheikhpour and Atia [26]	2019	Surveillance cameras, motion sensors	Device	Pose estimation accuracy	DCRNN	Calibration-free robust pose estimation, reducing localization error by 35% compared to traditional methods, with a mean error of less than 0.03 m in dynamic indoor environments, using deep convolutional recurrent neural networks
Dai et al. [27]	2020	Surveillance cameras, motion sensors	People	Accuracy and resilience	CNN, RNN	Accurate tracking in degraded environments with a positioning error under 0.5 m and a real-time processing rate of over 20 FPS using mmWave radar and IMU fusion
Hu and Liao [28]	2021	Sensor data, surveillance cameras	Device	Orientation error, position error, inference time	DNN	High accuracy and efficiency, achieving an orientation error of 2.5179° and a position error of 0.0222 m with an inference time under 4 ms per frame, supporting real-time camera localization in AR applications

## 5. Camera-Based Positioning with Radio Signals

Outdoor positioning is generally achieved through a radio interface that receives signals from one of the available GNSSs. A similar approach based on radio interfaces can be used indoors, based either on signal strength or timing measurements. In the former, a path loss model or radio map is used to assess the distance to a fixed anchor (such as Wi-Fi APs or Bluetooth beacons) based on the observed received signal strength (RSS) values. In the latter, mobile devices exchange data with the fixed anchors to calculate a relevant time metric (such as the time of flight) and infer the distance to these anchors. In the next step, distance estimates are combined using multilateration, which opens the way to indoor positioning. In this section, we provide examples of the integration of various wireless technologies within multimodal solutions to provide indoor localisation (as summarized in Table 3).

### 5.1. Localization Using Wi-Fi

Using Wi-Fi for localisation is an obvious choice considering both the availability of Wi-Fi networks (indoor deployments are already available in many buildings requiring navigation, such as shopping centres or airports) and the integration of Wi-Fi interfaces in modern devices (laptops and smartphones). All the papers reviewed in this section consider one type of Wi-Fi localisation—based on measuring the RSS values of Wi-Fi beacons and assuming that the radio signal attenuates only due to path loss (slow fading). Such values are called fingerprints and must be collected a priori during a dedicated measurement campaign (site survey) [2]. In addition to the cost of the site survey, this method is inexpensive as it does not require hardware modifications. Its accuracy, however, suffers in non-line of sight (NLOS) and fast fading conditions. RSS fingerprinting is not standardised and achieves a typical accuracy of 10–15 m, which can be improved as shown in the following papers.

Liu et al. [30] address indoor location for smartphone mobile users using a multisensor fusion approach. First, smartphone images are used as input for a deep CNN that provides scene recognition. The identified scene is then matched with a fingerprint database of Wi-Fi signals and magnetometer measurements. Additional smartphone built-in sensors (accelerometer, gyrometer, and compass) are used to determine the user’s heading. The proposed approach outperforms not only a similar system without scene recognition (that is, only with built-in sensors) but also a commercial indoor localisation platform, achieving a mean localization error in the evaluated real-world indoor office setting of only 1.32 m. A similar approach, i.e., using a CNN for camera image-based scene recognition and fusion with Wi-Fi RSS measurements, is proposed by Lin et al. [36].

Shao et al. [32] also rely on Wi-Fi and magnetic field measurements. However, instead of using cameras, they analyse the fingerprints as images, which is a surprising and rarely used approach. The high-resolution fingerprint images are constructed from a time series of sensor data. The authors design a custom CNN-based positioning model with separate branches to analyse Wi-Fi and magnetic field data, as well as a unifying branch. This CNN can automatically learn the relationship between the actual positions and the fingerprint images. The system was evaluated in an indoor office setting and achieved an accuracy of about 1 m regardless of smartphone orientations and use patterns.

Yan et al. [37] split the positioning problem into two stages: coarse and refined localisation. The first is done using RSS measurements of Bluetooth and cellular, long-term evolution (LTE) signals together with a support vector machine (SVM) trained to perform region classification. For the latter, Wi-Fi RSS fingerprints are transformed into images and combined with images taken from the smartphone’s camera, which then underwent a pyramidal decomposition. Next, a CNN provides the refined localisation estimate. The system was evaluated in an office setting, and the authors show that the proposed data fusion improves both the coarse localisation performance (in terms of the precision of the region classification) and the refined localisation (in terms of position error), the latter achieving an average error of below 0.5 m.

While the previous examples rely on smartphone cameras, Cheng et al. [39] use surveillance cameras combined with Wi-Fi signals to determine the positions of users in a room. They integrate an existing multi-person pose estimation system to extract foot placement using a novel algorithm. To reduce errors, this algorithm employs a variety of classic regression models (linear regression, Bayesian ridge, gradient-boosting regression). Wi-Fi signals, in the form of RSS values, are used to train the same models, with data taken simultaneously from two frequency bands (2.4 and 5 GHz). In an experimental setting, the entire system achieved an accuracy of below 0.5 m.

Wi-Fi fingerprinting can also support SLAM, which is crucial for autonomous robot navigation. Usman Shoukat et al. [40] present such an algorithm that fuses Wi-Fi signals (RSS) and images from the robot’s camera. Dynamic time warping (DTW) is used to match Wi-Fi fingerprint sequences while a YOLO algorithm detects objects. These data are joined in a graph SLAM algorithm, which provides an optimised trajectory for the robot. In a complex industrial environment, the proposed method achieved a positioning accuracy of about 3 m.

### 5.2. Localization Using UWB

Using UWB for indoor localisation has several important benefits: highly accurate time-of-arrival measurements, better wall penetration than other radio technologies, low energy use, and little interference with coexisting wireless systems [3]. Together, these benefits bring a localisation accuracy of even below 10 cm, but the main downside is the necessity of first installing dedicated UWB beacons. Furthermore, despite the penetration ability mentioned before, NLOS settings remain problematic. Below, we describe papers that use UWB as part of its multimodal indoor localisation scheme.

The first research paper in this area is by Nguyen et al. [29], who present the fusion of camera-based imagery with data from an UWB radio to improve localisation accuracy. In the proposed approach, vision data are used in two ways: (a) to reduce UWB’s positioning errors in NLOS settings and (b) to provide a cooperative positioning scheme. Regarding the former, since NLOS signals decrease the accuracy of UWB positioning, they need to be classified so that they can later be mitigated. Classification is done using SVM with a set of characteristics that typically consist of the energy received and the kurtosis of the received UWB waveform. The authors extend this set to include vision data, which reduces the identification error from 2.27% to 0.48%. The authors also present a cooperative positioning scheme: agents that can self-locate using camera imagery act as virtual anchors for other, nearby agents. This cooperation further reduces both the average network position error and the probability of outages.

A seamless positioning system is required when devices move from outdoor to indoor environments. Yang et al. [33] design such a system by combining GNSS data (outdoors) with UWB and Bluetooth data (indoors). This paper is one of the few examples that did not use camera images. Instead, the system converted the input data into a coordinate diagram, which is then analysed using a CNN. The evaluation is done with experiments in the transition region (moving from indoor to outdoor or vice versa), showing that the average positioning error is reduced by almost 50% to about 20 cm.

UWB is known to exhibit ranging errors in NLOS settings. Masiero et al. [34] (2020) address this issue by combining UWB measurements with images onboard the camera. The proposed system, installed on an autonomous robot that performs SLAM, uses deep learning to determine if a UWB anchor is visible. In addition to a standard camera, the robot also has a time of flight (ToF) camera, which measures depth. An experimental evaluation confirmed that the average positioning error can be reduced by at least 20%.

Indoor navigation is particularly challenging for groups of people operating and interacting in unknown environments, such as rescuers. Ruotsalainen et al. [35] develop a collaborative and infrastructure-free system that combines the data collected by each individual (camera images and UWB signals) and shares it among the group. A depth camera detects an object using a CNN, and an experimental trial demonstrated improvement over existing solutions. However, the authors mention several challenges, including the temporal lack of visual features, that still need to be addressed in such collaborative systems.

The final paper in this category, by Kao et al. [38] deals with indoor unmanned aerial vehicle (UAV) positioning. The proposed system integrates not only UWB and surveillance camera imagery but also data from the inertial sensors installed in a UAV. A CNN provides pose estimation; inertial characteristics are extracted using a long short-term memory (LSTM) network, while position estimation from UWB is done using a deep NN. In an experimental evaluation, the proposed data fusion approach provided the most consistent performance in pose estimation compared to other approaches.

### 5.3. Localization Using RFID

Indoor positioning is related not only to providing the location of mobile users but also to localising stationary items, which is crucial, e.g., in logistics. Berz et al. [31] design a multimodal system that adds camera imagery to an existing system based on radio-frequency identification (RFID). Each object is marked with an RFID tag and a visual marker, and both are initially analysed in separate subsystems. The first uses a NN model with RSS values as input, although an alternative SVM-based model is also proposed but performs worse. The second subsystem uses a k-means computer vision algorithm to find visual markers, and if the markers can be found, the output is fused with that of the first subsystem. Experiments in a lab setting showed that adding camera imagery improved performance by 32% compared to the RFID-only case, and the localisation precision is up to 9.1 cm.

## 6. Camera-Based Positioning with LiDAR

LiDAR is a remote sensing method that uses the properties of light (specifically, laser light) to measure distances with high precision by timing the interval between the emission of a laser pulse and the detection of its reflection from a target surface. This enables the construction of detailed three-dimensional representations of the scanned environment. LiDAR is highly useful for multimodal indoor localization for several reasons. LiDAR can offer high resolution for accurately modeling complex indoor spaces and capturing fine details of walls, furniture, and other objects. It provides accurate distance measurements by calculating the time it takes for a laser pulse to return after hitting an object. LiDAR systems can detect and map static and dynamic obstacles in real time, enhancing the ability of localization systems to adapt to changes in the environment, such as moving people or shifting furniture. LiDAR operates independently of ambient lighting conditions, making it effective in environments where cameras might struggle due to low light or glare. When used in a multimodal localization system, it can complement other sensors such as cameras, inertial measurement units (IMUs) and radio-based systems (Wi-Fi, Bluetooth).

### Key Advances in LiDAR-Based Multimodal Positioning

Significant advancements in semiconductor technology, MEMS, and optics led to the miniaturization of LiDAR systems in the 2010s. This period saw the integration of LiDAR into various applications, including drones, robotics, and smartphones [41].

One of the first attempts to introduce the combination of LiDAR and camera signals in the scope of multimodal indoor localisation is the work of Patel et al. [42]. It presents a novel framework for helping human operators in indoor navigation and map building. The framework allows a ground vehicle to learn navigation by imitating human steering commands, thus reducing the operator’s workload. The architecture integrates camera and LiDAR inputs to output the steering angle of the vehicle, including mechanisms to avoid static obstacles. The system can decide autonomously or transfer control to a human operator if it detects unreliable outputs. The paper demonstrates that the system can handle various conditions in which other frameworks may fail. The proposed method uses deep neural networks to process and merge data to accurately predict steering commands.

Ito et al. [43] concentrate on the capability of the new type of LiDAR device, namely single-photon avalanche diode (SPAD) LiDAR and a fusion-based localization method using a deep convolutional neural network (DCNN) for automated guided vehicles (AGVs). The SPAD LiDAR utilizes a time-of-flight method and SPAD arrays to output range image data, monocular image data, and peak intensity image data with the same coordinate system, eliminating the need for external calibration. The proposed SPAD DCNN method fuses these outputs to enhance the localization accuracy. The experiments were conducted in an indoor environment to positively evaluate the system’s performance under various AGV trajectories.

The next paper authored by Sun et al. [44] proposes a method to estimate the initial position of a monocular camera on a 3D LiDAR map without prior localisation information. This method uses an unsupervised CNN to predict depth images from monocular images and match them with depth maps projected from the LiDAR point cloud. The approach is evaluated using the KITTI dataset, demonstrating high precision in the estimation of initial positions. The framework is robust and versatile, showing satisfying results in various scenes without needing to retrain the network for different scenarios. However, one has to point out that while the authors claim that their goal is to provide indoor positioning, this scenario has not been tested (the KITTI dataset contains positioning data along with outdoor visual imaginary).

Jo and Kim [45] address a different challenge: improving the existing localization method with multimodal fusion. The proposed algorithm, called deep initialization, combines 3D LiDAR and camera data using a CNN to initialize particles for Monte Carlo localization (MCL). This approach addresses the limitations of traditional MCL methods that require long observation times and can suffer from localization failure. The effectiveness of the system is demonstrated through experiments on a mobile robot platform, showing improved localization accuracy and robustness. What is interesting is that the authors assessed the system’s ability to recover from localization failure and robot kidnapping scenarios.

The next study by Liu et al. [46] focuses on the location and navigation tasks of autonomous drones. A novel deep learning framework named visual-geometric fusion network (VGF-Net) is developed to combine visual and geometric information to enhance drone navigation and height mapping in complex environments. It is based on Res-Net architectures, and its output in the form of a visual-geometric representation is used in the Directional Attention Model to perform 3D localisation. In addition to outdoor scenarios, the authors evaluate their approach to indoor localisation for the S3DIS data set [47].

The work of Shuai and Yu [48] formulates another approach to improve the accuracy of the localisation of indoor mobile robots by combining monocular camera data and LiDAR technology. The method involves creating a training dataset by projecting a 3D point cloud map of the known environment onto a grid map, capturing images with a monocular camera, and assigning coordinates to each image. A deep learning algorithm, based on CNNs, is then used to train a model that can determine the camera’s location from 2D RGB images. The estimated coordinates are refined using LiDAR data to achieve more precise localisation. This fusion approach improves positioning accuracy and efficiency, providing better support for robot indoor navigation and operations.

Chen and Hong [49] contribution focuses on improving the perception system of quadruped robots using a combination of LiDAR and vision sensors. The study compares single-sensor SLAM methods using either LiDAR or vision sensors and then explores multisensor SLAM to enhance environmental perception. Here an extended Kalman filter is being employed for the fusion itself. Additionally, the paper incorporates an improved YOLOv5 network with adaptive spatial feature fusion (ASFF) for gesture recognition, enabling human-robot interaction. The research findings indicate that multisensor fusion significantly improves mapping and localization accuracy and robustness in complex environments.

The following two studies enrich the perspective of multimodal LiDAR-based data fusion by incorporating the signals from inertial sensors. The paper by Cai et al. [50] integrates data from inertial measurement units (IMUs), LiDAR, and cameras using the discrete Kalman filter (DKF) and extended Kalman filter (EKF) principles. The fusion scheme involves a three-step joint filtering model that processes visual data with the YOLO deep learning target detection algorithm to enhance state estimation. The proposed method achieves high precision positioning with a low three-dimensional root mean square error (RMSE) effectively suppressing motion errors in occluded environments. The work of Tian et al. [51] also includes the addition of a wheel encoder signal. It proposes an improved grid mapping algorithm [52] designed to enhance the SLAM capabilities of robots. The improvement is based on the addition of the deep learning-based loop detection method for better map consistency. For this purpose, a combination of two CNNs is used (AlexNet and Mobilenet v3-Large). In addition to a positive evaluation with the original GMapping algorithm, the authors study different fusion schemes (e.g., using only LiDAR end encoder data or laser, encoder, and IMU data). They summarize that a complete set of multimodal signals is very beneficial for obtaining accurate SLAM.

Finally, a recent paper by Wong et al. [53] presents a novel approach to enhance localisation accuracy and stability for mobile robots in complex indoor environments with the use of LiDAR, RGB-D cameras, motor encoders, and advanced algorithms such as deep reinforcement learning (DRL) and adaptive multi-model estimation (MMAE). In the two simulated cases, their algorithm performed better in terms of accuracy and robustness than unimodal strategies based only on LiDAR or image processing.

Table 4 covers advances in LiDAR-based applications of multimodal indoor positioning methods identified in our study.

## 7. Camera-Based Positioning with Other Sensors

The final category of papers concerns fusing image data with sensors such as sound, magnetic field, or chemical (e-nose) data inputs. It is not a very common approach, as only three papers of this kind were identified. They are summarized in Table 5.

The paper by Monroy et al. [54] presents a novel approach for localizing gas emission sources in indoor environments using a mobile robot equipped with vision and chemical sensors. The system fuses data from these sensors to leverage the semantic relationships between detected gases and visually recognized objects, improving the efficiency and accuracy of gas source localization. The approach uses a probabilistic Bayesian framework to handle uncertainties in gas classification and object recognition, dynamically updating the list of potential gas source candidates. A Markov decision process based path planning algorithm is employed to minimize the search time by prioritizing candidate objects based on their probabilities and distances.

Study of Yan et al. [55] introduces the memory vision-voice indoor navigation (MVV-IN) system, which enhances indoor navigation by integrating vision and voice commands. The MVV-IN system uses a monocular camera to capture RGB images and employs a self-attention mechanism to focus on key areas, while also incorporating meta-learning to adapt to new scenes and avoid redundant tasks. The proposed method demonstrates significant improvements in navigation performance compared to state-of-the-art baselines through extensive experiments.

Finally, an interesting approach is discussed in the work of Opiela et al. [56]. Not only does it use a magnetic field sensor along with camera input, but it also employs two different machine learning approaches—LSTM and CNN. It also transforms the localisation problem into a classification task, with the building parts being accurately recognized.

## 8. Summary and Open Challenges

We have reviewed the literature in the area of indoor localization, where positioning is achieved through ML-based integration of multimodal information, including image data. Our review clearly indicates the significant advances that indoor positioning systems have made in recent years thanks to the integration of multiple data sources such as camera imagery, motion sensors, radio interfaces, and LiDAR measurements. We summarize our findings below by providing broad conclusions, identifying open research areas, as well as commenting on the future of indoor positioning systems.

In terms of general conclusions, we first note that all the presented multimodal approaches improve accuracy (i.e., reduce errors) of indoor positioning. In particular, research clearly shows that the multimodal approach outperforms a unimodal one. Thus, we expect that future indoor positioning systems will combine at least two input modalities. Second, we observe that ML is particularly useful in this area. Machine learning methods for indoor localization using sensor fusion often leverage CNNs and deep CNNs to integrate data from multiple sensors. These methods use CNNs to extract features from visual data, such as RGB images, and combine them with depth or range data from sensors like LiDAR to improve localization accuracy. By embedding the features from different modalities into a unified representation, the models enhance environmental perception, allowing for more accurate navigation and obstacle detection. Furthermore, deep learning architectures such as YOLO, enhanced with adaptive spatial feature fusion, are used for real-time object detection and semantic interpretation, further supporting localization by identifying key objects and features in the environment. These networks are trained in an end-to-end manner to predict positional information or make navigational decisions based on the fused sensory data, ensuring robust performance in complex indoor environments. As our third general conclusion, we note that an overwhelming majority of research was done experimentally (i.e., in real-life testbeds), which is commendable. Analytical or simulation studies would be overly simplified for studying the accuracy of indoor positioning. The research is also convergent with respect to localized objects (mainly devices such as smartphones) and metrics used (predominantly the root mean square error of the estimated position).

Despite the described advancements, we can still identify several general research challenges. First, privacy concerns are paramount due to the widespread use of cameras in public and private spaces. Ensuring these systems operate without compromising user privacy and securely handling data is paramount to gaining user trust and regulatory approval. We have not seen such issues mentioned in the surveyed papers but foresee that implementing advanced encryption techniques, developing privacy-preserving data processing algorithms, and establishing clear guidelines for data usage and storage are necessary steps in this area.

Another research challenge is the appropriate selection of inputs. We believe researchers should focus on integrating data from readily available sources. In the case of smartphones, these include motion sensors, built-in cameras, and radio interfaces (Wi-Fi and Bluetooth).

Furthermore, researchers in this field should exploit the advances in artificial intelligence, particularly deep learning, to create more intelligent and adaptive positioning systems. ML models that continuously learn from new data in real time could handle dynamic indoor environments more effectively, including such features as moving obstacles or changing lighting conditions. In this aspect, we foresee the necessity for interdisciplinary collaboration among experts in computer vision, robotics, and wireless communication to drive the development of more sophisticated positioning systems. Integrating advanced computer vision techniques with robotic navigation algorithms could lead to highly accurate and autonomous indoor navigation systems.

Another underexplored area of research is the reduction of power consumption. Exploring energy-efficient algorithms and hardware implementations is essential for widespread adoption in portable devices. Reducing power consumption without compromising performance is a key to making positioning systems more practical for continuous use in mobile and wearable applications. Researchers should take energy efficiency into account in their designs. However, we can also expect the near-future development of specialised hardware accelerators for machine learning models and the optimisation of existing ML algorithms to run efficiently on low-power devices.

An additional research challenge is the evaluation of proposed solutions, which are typically done in single environments. Meanwhile, testing in real-world environments such as shopping malls, airports, and industrial sites is crucial. Such extensive large-scale field trial tests will provide valuable insight into the performance and scalability of these systems, identifying potential challenges and areas for improvement.

Our final identified general open research area pertains to reproducibility: a key component of the advancement of indoor positioning research is the availability of diverse datasets for training and evaluation. We have analysed the papers to identify the datasets used therein. Most of the contributions use proprietary datasets that are not enclosed for replicability. Table 6 summarises the datasets used in the surveyed literature, highlighting the variety and scope of data that has been instrumental in the development and benchmarking of these systems. It is our important observation that future research should continue to leverage and expand such datasets, ensuring that they cover a wide range of scenarios and environments to improve the generalisability and robustness of positioning solutions.

We also identify three technology-specific open research challenges. First, there is a need for more robust and reliable camera systems capable of operating under various conditions, such as low light and extreme temperatures. Second, integrating existing camera sensors with complimentary technologies like LiDAR and radar can further enhance their capabilities in terms of indoor positioning. Third, we have not identified any multimodal indoor positioning system that would consider Wi-Fi Location, which is based on the fine timing measurement (FTM) feature of the IEEE 802.11 standard, which is an active point-to-point protocol based on time for positioning, first proposed in IEEE 802.11 REVmc and later extended in IEEE 802.11az and IEEE 802.11bk.

We conclude by looking at the future of indoor positioning systems. Integrating these systems into consumer products, such as smartphones and wearable devices, can extend their utility, improve user experiences in everyday applications, and pave the way for new applications and functionalities in healthcare, logistics, and entertainment. Advanced indoor positioning systems can aid visually impaired individuals, provide precise tracking for fitness applications, or enable immersive gaming experiences. We maintain that the integration of deep learning and computer vision with camera sensors opens new opportunities for research and development, paving the way for future innovations. The journey towards achieving precise indoor localisation continues, with ongoing research addressing current challenges and exploring new directions for future advancements.

## Figures and Tables

**Table 1:** Summary of camera-based sensor studies.

Reference	Year	Input Data	Localized Object	Metrics	ML Method	Outcome
Petrushin et al. [19]	2005	Web cameras, infrared badge ID system, PTZ cameras	People	Precision, recall	Bayesian inference, SOM	Enhanced surveillance with multiple sensors, achieving a precision of 87.21% and a recall of 73.55% for people localization through sensor fusion
Md Mahfujur Rahman and El Saddik [17]	2011	Cameras	Device, maps	Engagement, usability	None	Interactive learning experiences enhanced through real-world object annotations, improving user engagement by 30% in educational gaming scenarios, particularly among younger users
Narayanan et al. [18]	2013	Cameras (Omnidirectional)	Robot	Navigation accuracy, obstacle avoidance	GMM	Learns, generalizes behaviors, enabling robust navigation and obstacle avoidance with a success rate of 100% across varying indoor environments using Gaussian mixture models
Bai et al. [13]	2019	Cameras	Device	Pose accuracy	Various deep learning models	Accurate positioning and navigation improved through deep learning models, with pose accuracy enhanced to within centimeters for indoor environments
Narayanan et al. [14]	2020	Cameras	People	Emotion prediction precision	Conv. model	Social navigation enhanced with emotion prediction precision of 82.47%, leading to safer and more socially-aware robot paths in crowded environments
Zhang and Leonard [15]	2021	Cameras	Object	Pose accuracy, robustness	CNN	Improved mapping with pose accuracy enhanced by 28% and robust depth estimation under dynamic object interference using a learned outlier mask for better occlusion handling
Yang et al. [16]	2022	Cameras	Env.	Point cloud accuracy	CNN	Better point cloud reconstruction with a point cloud accuracy of 90%, reducing absolute trajectory error from 0.243 m to 0.037 m in indoor scenes with rich feature points
Wen et al. [20]	2023	Cameras	Navigation agents	Navigation success, trajectory length	A2C, attention	Better navigation and planning achieved, with success rates improved by 5.3% and navigation trajectory length reduced by 8.2% using cross-modal feature fusion in unknown environments

**Table 3:** Summary of works on radio-based positioning.

Reference	Year	Input Data	Localized Object	Metrics	ML Method	Outcome
Nguyen et al. [29]	2014	Camera, UWB	Device	Localization error	SVM, VMP	Vision-radio fusion reduces ranging error by at least 2 m under NLOS conditions.
Liu et al. [30]	2017	Camera, WiFi, inertial and magnetic field sensors	Smartphone	Localization error	CNN, PF	Improved location accuracy (1.32 m at 95%) through scene (room) recognition.
Berz et al. [31]	2017	Camera, RFID	Object	Localization error	ANN, SVR, k-means	Improved 2D localization of objects (error below 30 cm at a distance of 2 m) compared to RFID-only case.
Shao et al. [32]	2018	Wi-Fi, inertial sensors	Smartphone	Localization error	CNN	CNN automatically learns location patterns for a positioning accuracy of 1 m.
Yang et al. [33]	2020	UWB, Bluetooth, GNSS	Device	Localization error	CNN	Improved positioning accuracy (by 50%) in outdoor/indoor transition regions.
Masiero et al. [34]	2020	Camera, UWB	Device	Localization error	Deep learning-based classifier	UWB ranging errors reduced by 20% in NLOS settings.
Ruotsalainen et al. [35]	2022	Camera, UWB, inertial sensors	Device	Localization error	KF, CNN	Collaborative, infrastructure-free system for rescuers (with incremental accuracy improvement from visual odometry).
Lin et al. [36]	2022	Camera, Wi-Fi	Device	Region-based localization accuracy	CNN	Feature extraction from both image and RSS data improves zone-based localization up to 93%.
Yan et al. [37]	2023	Camera, Wi-Fi, LTE, Bluetooth	Device	Region-based localization accuracy	CNN	RSS fingerprints combined with camera data reduce positioning error (below 1 m at 90th percentile).
Kao et al. [38]	2023	Camera, UWB, inertial sensors	UAV	Localization error (translation)	CNN, LTSM, deep NN	UAV localization improved through pose estimation from external cameras, fusion method has lowest global 3D translation error.
Cheng et al. [39]	2023	Camera, Wi-Fi	Smartphone	Distance error	linear regression, Bayesian ridge, gradient boosting regression	User positioning (with average error of 0.43 m) through feet placement extraction from surveillance cameras.
Usman Shoukat et al. [40]	2024	Camera, Wi-Fi	Robot	Localization error	Deep NN	SLAM system for autonomous robots with at least 20% higher accuracy than baseline.

**Table 4:** Summary of LiDAR-based applications of multimodal indoor positioning methods.

Reference	Year	Input Data	Localized Object	Metrics	ML Method	Outcome
Patel et al. [42]	2017	Camera, LiDAR	Robot	Accuracy of localization	CNNs	Accurate navigation through an indoor environment. Time domain graphs were compared without summary metrics.
Ito et al. [43]	2018	LiDAR (3 inputs)	Vehicle	Accuracy of localization	CNNs	Fusing multiple camera inputs with new LiDAR module allowed to reduce localisation error by 30%.
Sun et al. [44]	2019	Camera, LiDAR	Device	Precision and Recall (localization)	CNNs	Accurate localization—improvement of shape of precision-recall curves—with multimodal representation of places
Jo and Kim [45]	2020	Camera, 3D LiDAR	Robot	Accuracy of localization	CNNs	Improved localization with Monte Carlo Localization (MCL) algorithm. Obtained over 50% RMSE reduction with the introduced Deep initialisation strategy.
Liu et al. [46]	2021	Camera, LiDAR	Drone	Accuracy of Height map; Navigation success	CNNs	Fusing visual and geometric information for accurate navigation—over 10% navigation success rate improvement for S3DIS dataset.
Shuai and Yu [48]	2021	Camera, LiDAR	Robot	Accuracy of localization	CNNs	Improved positioning accuracy with multimodal input, when compared with single sensor approach.
Chen and Hong [49]	2023	Camera, LiDAR	Robot	Mapping quality	Extended Kalman Filter, CNNs	High performance of the environmental perception—visual maps provided.
Cai et al. [50]	2023	Camera, LiDAR, inertial sensors	Robot	Accuracy of localization	YOLO and Kalman filter	Low-cost and robust multi-sensor localization with multi-robot collaborative navigation—trajectories provided.
Tian et al. [51]	2023	Camera, LiDAR, inertial sensors, wheel encoder	Robot	Mapping quality and location accuracy	CNNs	Robust and precise localization with the improved Gmapping—visual maps provided.
Wong et al. [53]	2024	Camera, LiDAR, IMU, motor encoder	Robot	Accuracy of localization	CNNs, Reinforcement Learning	Higher accuracy and localization stability than traditional methods. Performance improvement for more than 70% test scenarios.

**Table 5:** Summary of contributions to camera-based positioning with other sensors.

Reference	Year	Input Data	Localized Object	Metrics	ML Method	Outcome
Monroy et al. [54]	2018	Camera, chemical sensors	Robot, event	Event detection	CNNs	Robot learns semantic relationships among the detected gases and the objects visually recognized. Real experiments with objects and gas sources distributed along the path.
Yan et al. [55]	2020	Camera, microphone	Agent	Navigation success ratio	CNNs	Using voice commands and images to enhance robots’ environment understanding. Both qualitative and quantitative data provided, with the improvement of path lengths also observed.
Opiela et al. [56]	2023	Camera, magnetic field sensors	Smartphone	Location classification accuracy	LSTM, CNNs	Classifying building parts with LSTM on magnetic field data, and CNN network on camera images. Improvement of approx. 0.1 in F1-score values.

**Table 6:** Datasets used in the surveyed literature.

Name	Used in	Description
VIO	Soroush Sheikhpour and Atia [26]	Dataset for testing the robustness of various VO/VIO methods, acquired on a UAV. Contains 4 trajectories, together with image and IMU data.
EuRoC	Kao et al. [38]	Dataset comprised of 11 sequences with synchronized stereo images, IMU measurements, and ground-truth poses.
S3DIS	Liu et al. [46]	Indoor scene dataset which contains 6 large-scale indoor areas with 271 rooms. Each point in the scene point cloud is annotated with one of the 13 semantic categories.
KITTI	Zhang and Leonard [15], Sun et al. [44]	Outdoor dataset captured while driving around the mid-size city containing data from two high-resolution color and grayscale video cameras and ground truth from laser scanner and a GPS.

## Data Availability

Data are contained within the article.

## Abbreviations

The following abbreviations are used in this manuscript:

A2C	advantage actor-critic
AP	access point
AR	augmented reality
CCD	charge-coupled device
CNN	convolutional neural network
DCNN	deep convolutional neural network
DCRNN	deep convolutional recurrent neural network
DTW	dynamic time warping
FTM	fine timing measurement
GIS	geographic information system
GMM	Gaussian mixture model
GNSS	global navigation satellite system
GPS	global positioning system
ID	identifier
IMU	inertial measurement unit
IR	infrared
KF	Kalman filter
LBS	location-based service
LiDAR	light detection and ranging
LSTM	long short-term memory
LTE	long-term evolution
MEMS	micro-electro-mechanical system
ML	machine learning
MSER	maximally stable extremal regions
MSIS	multiple sensor indoor surveillance
NLOS	non-line of sight
NN	neural network
PDR	pedestrian dead reckoning
PF	particle filter
PTZ	pan-tilt-zoom
RFID	radio-frequency identification
RGB	red-green-blue
RGB-D	red-green-blue-depth
RMSE	root mean square error
RSS	received signal strength
SDF-SLAM	signed distance function SLAM
SIFT	scale-invariant feature transform
SLAM	simultaneous localization and mapping
SOM	self-organising map
SVM	support vector machine
ToF	time of flight
UAV	unmanned aerial vehicle
UWB	ultra-wideband
VLN	vision and language navigation

## References

[1] Merry K., Bettinger P. (2019). Smartphone GPS accuracy study in an urban environment. PLoS ONE.

[2] Dai J., Wang M., Wu B., Shen J., Wang X. (2023). A Survey of Latest Wi-Fi Assisted Indoor Positioning on Different Principles. Sensors.

[3] Leitch S.G., Ahmed Q.Z., Abbas W.B., Hafeez M., Laziridis P.I., Sureephong P., Alade T. (2023). On Indoor Localization Using WiFi, BLE, UWB, and IMU Technologies. Sensors.

[4] Narasimman S.C., Alphones A. (2024). DumbLoc: Dumb Indoor Localization Framework Using Wi-Fi Fingerprinting. IEEE Sensors J..

[5] Bi J., Wang J., Cao H., Yao G., Wang Y., Li Z., Sun M., Yang H., Zhen J., Zheng G. (2024). Inverse distance weight-assisted particle swarm optimized indoor localization. Appl. Soft Comput..

[6] Liu Q., Zhao Y., Yin Z., Wu Z. (2024). WDMA-UWB Indoor Positioning through Channel Classification-Based NLOS Mitigation Approach. IEEE Sensors J..

[7] Wang F., Shui L., Tang H., Wei Z. (2024). Enhancing UWB Indoor Positioning Accuracy through Improved Snake Search Algorithm for NLOS/LOS Signal Classification. Sensors.

[8] Grega M., Matiolański A., Guzik P., Leszczuk M. (2016). Automated detection of firearms and knives in a CCTV image. Sensors.

[9] Krzywda M., Łukasik S., Gandomi A.H. (2022). Graph neural networks in computer vision-architectures, datasets and common approaches. Proceedings of the 2022 International Joint Conference on Neural Networks (IJCNN).

[10] Szott S., Kosek-Szott K., Gawłowicz P., Gómez J.T., Bellalta B., Zubow A., Dressler F. (2022). Wi-Fi Meets ML: A Survey on Improving IEEE 802.11 Performance With Machine Learning. IEEE Commun. Surv. Tutorials.

[11] Pollock A., Berge E. (2018). How to do a systematic review. Int. J. Stroke.

[12] Mendoza-Silva G.M., Torres-Sospedra J., Huerta J. (2019). A meta-review of indoor positioning systems. Sensors.

[13] Bai X., Huang M., Prasad N.R., Mihovska A.D. A Survey of Image-Based Indoor Localization using Deep Learning. Proceedings of the International Conference on Intelligent Computing and Internet of Things (ICIT).

[14] Narayanan V., Manoghar B.M., Sashank Dorbala V., Manocha D., Bera A. ProxEmo: Gait-based emotion learning and multi-view proxemic fusion for socially-aware robot navigation. Proceedings of the IEEE International Conference on Robotics and Automation (ICRA).

[15] Zhang Y., Leonard J.J. A Front-End for Dense Monocular SLAM using a Learned Outlier Mask Prior. Proceedings of the IEEE Vehicle Power and Propulsion Conference (VPPC).

[16] Yang C., Chen Q., Yang Y., Zhang J., Wu M., Mei K. (2022). SDF-SLAM: A Deep Learning Based Highly Accurate SLAM Using Monocular Camera Aiming at Indoor Map Reconstruction With Semantic and Depth Fusion. IEEE Access.

[17] Md Mahfujur Rahman A.S., El Saddik A. Mobile pointme based pervasive gaming interaction with learning objects annotated physical atlas. Proceedings of the IEEE International Conference on Multimedia and Expo (ICME).

[18] Narayanan K., Posada L., Hoffmann F., Bertram T. Acquisition of behavioral dynamics for vision based mobile robot navigation from demonstrations. Proceedings of the IFAC Mechatronics Symposium.

[19] Petrushin V.A., Wei G., Ghani R., Gershman A.V. Multiple sensor integration for indoor surveillance. Proceedings of the Sixth International Conference on Spoken Language Processing (ICSLP 2000).

[20] Wen S., Lv X., Yu F.R., Gong S. (2023). Vision-and-Language Navigation Based on Cross-Modal Feature Fusion in Indoor Environment. IEEE Trans. Cogn. Dev. Syst..

[21] Yan J., He G., Basiri A., Hancock C. Indoor pedestrian dead reckoning calibration by visual tracking and map information. Proceedings of the IEEE International Conference on Communications (ICC).

[22] Turaga P., Ivanov Y.A. (2011). Diamond sentry: Integrating sensors and cameras for real-time monitoring of indoor spaces. IEEE Sensors J..

[23] Yan J., He G., Basiri A., Hancock C. Vision-aided indoor pedestrian dead reckoning. Proceedings of the IEEE International Conference on Communications (ICC).

[24] Xu Y., Yu H., Zhang J. (2018). Fusion of inertial and visual information for indoor localisation. Electron. Lett..

[25] Li X., Sridharan M. Safe navigation on a mobile robot using local and temporal visual cues. Proceedings of the International Conference on Computer Science and Information Technology (ICCSIT).

[26] Soroush Sheikhpour K., Atia M.M. Calibration-free visual-inertial fusion with deep convolutional recurrent neural networks. Proceedings of the International Conference on Information and Communication Technology (ICICT).

[27] Dai Z., Saputra M.R.U., Lu C.X., Trigoni N., Markham A. Indoor positioning system in visually-degraded environments with millimetre-wave radar and inertial sensors: Demo abstract. Proceedings of the International Conference on Computer and Information Technology (ICCIT).

[28] Hu T., Liao Q. Real-Time Camera Localization with Deep Learning and Sensor Fusion. Proceedings of the IEEE International Conference on Networks (ICN).

[29] Nguyen T., Jeong Y., Trinh D., Shin H. (2014). Location-aware visual radios. IEEE Wirel. Commun..

[30] Liu M., Chen R., Li D., Chen Y., Guo G., Cao Z., Pan Y. (2017). Scene recognition for indoor localization using a multi-sensor fusion approach. Sensors.

[31] Berz E.L., Tesch D.A., Hessel F.P. A hybrid RFID and CV system for item-level localization of stationary objects. Proceedings of the International Conference on Pattern Recognition (ICPR).

[32] Shao W., Luo H., Zhao F., Ma Y., Zhao Z., Crivello A. (2018). Indoor Positioning Based on Fingerprint-Image and Deep Learning. IEEE Access.

[33] Yang G., Zhang X., Zhu S., Zhang J. Convolutional Neural Network based UWB/BLE/BDS Fusion Positioning System. Proceedings of the IEEE International Conference on Security and Privacy (ICSP).

[34] Masiero A., Perakis H., Gabela J., Toth C., Gikas V., Retscher G., Goel S., Kealy A., Koppányi Z., Błaszczak-Bak W., Paparoditis N., Mallet C., Lafarge F., Hinz S., Feitosa R., Weinmann M., Jutzi B. (2020). Indoor navigation and mapping: Performance analysis of UWB-based platform positioning. Proceedings of the XXIV ISPRS Congress.

[35] Ruotsalainen L., Morrison A., Makela M., Rantanen J., Sokolova N. (2022). Improving Computer Vision-Based Perception for Collaborative Indoor Navigation. IEEE Sensors J..

[36] Lin L., Yang L., Dong W., Yang S., Yu B. A Feature Extration Method based on Bi-Tower for Indoor Positioning. Proceedings of the IEEE Global Communications Conference (GLOBECOM).

[37] Yan J., Huang Z., Wu X. (2023). Smartphone Based Indoor Localization Using Machine Learning and Multi-Source Information Fusion. IEEE Trans. Aerosp. Electron. Syst..

[38] Kao P.Y., Chang H.J., Tseng K.W., Chen T., Luo H.L., Hung Y.P. (2023). VIUNet: Deep Visual-Inertial-UWB Fusion for Indoor UAV Localization. IEEE Access.

[39] Cheng H.H., Leu J.S., Liu J.X. (2023). Two-Phase Positioning System Based on the Fusion of Wi-Fi Signal Strength and Pose Estimation. IEEE Syst. J..

[40] Usman Shoukat M., Yan L., Deng D., Imtiaz M., Safdar M., Ali Nawaz S. (2024). Cognitive robotics: Deep learning approaches for trajectory and motion control in complex environment. Adv. Eng. Inform..

[41] Neff T. (2018). The Laser That’s Changing the World: The Amazing Stories behind Lidar, from 3D Mapping to Self-Driving Cars.

[42] Patel N., Krishnamurthy P., Fang Y., Khorrami F. Reducing operator workload for indoor navigation of autonomous robots via multimodal sensor fusion. Proceedings of the International Conference on Computing and Network Communications (CoCoNet).

[43] Ito S., Hiratsuka S., Ohta M., Matsubara H., Ogawa M. (2018). Small imaging depth LIDAR and DCNN-based localization for automated guided vehicle. Sensors.

[44] Sun M., Yang S., Liu H. (2019). Convolutional neural network-based coarse initial position estimation of a monocular camera in large-scale 3D light detection and ranging maps. Int. J. Adv. Robot. Syst..

[45] Jo H., Kim E. (2020). New Monte Carlo Localization Using Deep Initialization: A Three-Dimensional LiDAR and a Camera Fusion Approach. IEEE Access.

[46] Liu Y., Xie K., Huang H. (2021). VGF-Net: Visual-Geometric fusion learning for simultaneous drone navigation and height mapping. Graph. Model..

[47] Armeni I., Sener O., Zamir A., Jiang H., Brilakis I.K., Fischer M., Savarese S. 3D Semantic Parsing of Large-Scale Indoor Spaces. Proceedings of the 2016 IEEE Conference on Computer Vision and Pattern Recognition (CVPR).

[48] Shuai Z., Yu H. Multi-sensor fusion for autonomous positioning of indoor robots. Proceedings of the International Conference on Computer and Communication Systems (ICCCS).

[49] Chen G., Hong L. (2023). Research on Environment Perception System of Quadruped Robots Based on LiDAR and Vision. Drones.

[50] Cai Z., Liu J., Chi W., Zhang B. (2023). A Low-Cost and Robust Multi-Sensor Data Fusion Scheme for Heterogeneous Multi-Robot Cooperative Positioning in Indoor Environments. Remote. Sens..

[51] Tian C., Liu H., Liu Z., Li H., Wang Y. (2023). Research on Multi-Sensor Fusion SLAM Algorithm Based on Improved Gmapping. IEEE Access.

[52] Grisetti G., Stachniss C., Burgard W. (2007). Improved Techniques for Grid Mapping with Rao-Blackwellized Particle Filters. IEEE Trans. Robot..

[53] Wong C.C., Feng H.M., Kuo K.L. (2024). Multi-Sensor Fusion Simultaneous Localization Mapping Based on Deep Reinforcement Learning and Multi-Model Adaptive Estimation. Sensors.

[54] Monroy J., Ruiz-Sarmiento J.R., Moreno F.A., Melendez-Fernandez F., Galindo C., Gonzalez-Jimenez J. (2018). A semantic-based gas source localization with a mobile robot combining vision and chemical sensing. Sensors.

[55] Yan L., Liu D., Song Y., Yu C. Multimodal aggregation approach for memory vision-voice indoor navigation with meta-learning. Proceedings of the 2020 IEEE/RSJ International Conference on Intelligent Robots and Systems (IROS).

[56] Opiela M., Stedlová V.M., Horvát S., Antoni L., Hajduková L. Building Parts Classification using Neural Network. Proceedings of the CEUR-WS 2023.

